# Comprehensive analysis of tertiary lymphoid structures-related genes for prognostic prediction, molecular subtypes and immune infiltration in gastric cancer

**DOI:** 10.18632/aging.205247

**Published:** 2023-11-27

**Authors:** Qingde Zhou, Lan Lan, Wei Wang, Xinchang Xu, Wei Wang

**Affiliations:** 1 Department of Pharmacy, Hangzhou Third People’s Hospital, Hangzhou Third Hospital Affiliated to Zhejiang Chinese Medical University, Affiliated Hangzhou Dermatology Hospital, Zhejiang University School of Medicine, Hangzhou 310009, People’s Republic of China; 2 Department of Dermatology, Affiliated Hangzhou Dermatology Hospital, Zhejiang University School of Medicine, Hangzhou Third People’s Hospital, Hangzhou 310009, People’s Republic of China

**Keywords:** gastric cancer, tertiary lymphoid structures, molecular subtype, nomogram, immune landscape

## Abstract

Gastric cancer (GC) is a highly heterogeneous malignancy and survival rates of advanced GC patients are unsatisfactory. Tertiary lymphoid structures (TLS) are recently identified as lymphoid-like structures that are directly related to tumor prognosis and immune response. However, the association of tertiary lymphoid structures-related genes (TLS-RGs) with prognosis and immune response in GC remains unclear. In our study, a comprehensive analysis of the role of TLS-RGs in GC was performed based on public data, and the difference of TLS-RGs expression, TLS-RGs mutation frequency, pathway enrichment, differentially expressed gene, immune landscape, immunotherapy and drug sensitivity was analyzed. We found that TLS-RGs were altered in GC in terms of expression and mutation. The difference of survival, immune landscape and enrichment pathway exists between TLS clusters. Immune checkpoint differences were also evident between gene clusters. The grouping by TLS score indicated that patients in the low TLS score group had a better prognosis and a lower degree of immune escape. For immunotherapy, the low TLS score group showed better outcomes than the high TLS score group. Sensitivity to chemotherapeutic agents differed between TLS score groups. In conclusion, we comprehensively analyzed the role of TLS-RGs in GC, constructed nomogram that can accurately predict the prognosis of GC patients, and the TLS score can reflect the immune landscape of patients, providing the possibility of personalized design of immunotherapy and targeted drug therapy for GC patients.

## INTRODUCTION

Gastric cancer (GC) is a malignant tumor caused by uncontrolled growth of epithelial cells in the gastric mucosa [1, 2]. According to statistics, there will be more than 1 million new GC cases and 800,000 GC death cases in worldwide in 2020 alone [3]. It is estimated that in the next 25 years, there will be an additional 10 million GC cases and nearly 6 million deaths from gastric cancer [4]. Early GC is usually not clinically symptomatic, which leads to advanced disease once diagnosed [5, 6]. Surgical treatment remains the most common treatment for advanced GC. However, due to the specificity and metastatic nature of GC, the median survival rate of patients with advanced GC patients is less than one year [7, 8]. The cure rate for metastatic GC remains low even with the advent of new targeted drugs and immunotherapy [9, 10]. Therefore, early diagnosis and treatment of GC is the key to saving the lives of GC patients.

Tertiary lymphoid structures (TLS) have recently been identified as a class of lymphoid structures, and studies suggest that it forms only in a variety of chronic inflammatory diseases, autoimmune conditions, and cancers [11, 12]. Notably, the presence of TLS correlates with tumor prognosis and the response to immunotherapy [13, 14]. The prognostic value of TLS has been specifically studied in a variety of tumors, including intrahepatic cholangiocarcinoma [15], endometrial cancer [16], oral cavity cancer [17], lung cancer [18] and renal clear cell carcinoma [19], among others. These studies not only demonstrated the importance of TLS on the prognosis of tumor patients, but highlighted the importance of TLS affecting immune infiltration in improving patient survival. Immunotherapy has been shown to be associated with a variety of tumor treatments [20, 21], and TLS is involved in the formation of tumor immunity and largely influences the outcome of immunotherapy [22]. Tumor immunity is also the key to the treatment of GC. Therefore, focusing on the role of TLS in GC, especially on the alteration of tertiary lymphoid structures-related genes (TLS-RGs) in GC patients and the identification of TLS-RGs subgroups, may improve the poor prognostic outcome of GC patients.

In our study, the expression and mutation of TLS-RGs in GC patients were analyzed. GC patients were divided into different TLS clusters based on TLS-RGs and the differences in survival time, signaling pathways, immune landscape and expression profiles between them were analyzed. Based on the co-differentially expressed genes among TLS clusters, GC patients were divided into different gene clusters and the differences in survival time, expression profiles, and immune checkpoints were analyzed. The GC patients were divided into high or low TLS score groups based on TLS scores. Nomogram with satisfactory predictive accuracy was constructed. We also systematically analyzed the effects of TLS-RGs on tumor immunity, immunotherapy response, drug sensitivity, and tumor mutation burden in GC patients by multiple algorithms. In conclusion, our study comprehensively described the prognostic and predictive value of TLS-RGs in GC. In particular, focusing on the role of TLS-RGs in regulating tumor immunity will help to fully explore TLS as a potential target for GC and improve the prognosis of GC patients.

## MATERIALS AND METHODS

### Download of GC dataset and acquisition of TLS-RGs

The RNA expression data, clinical characteristics information, and somatic mutation data about GC were downloaded from the The Cancer Genome Atlas (TCGA) database (https://portal.gdc.cancer.gov/). The GC-related dataset GSE84437 with complete clinical information and sufficient number of samples was located and downloaded from the GEO database (https://www.ncbi.nlm.nih.gov/geo/). Those samples without complete information were excluded, and the gene ID-transformed datasets were batch corrected and merged by the “sva” package and the “Combat” package, and the merged datasets were used for subsequent analysis. 39 TLS-RGs were obtained from previous studies [23]. The clinical information and data involved in this study were obtained from public databases. Therefore, written informed consent from patients and approval from the ethics committee were not required for this study.

### Differential expression analysis and mutation analysis

To identify DEGs between normal and GC samples or between different subgroups, differential analysis was performed by the “limma” package based on |foldchange| ≥ 1 and *P* < 0.05. Mutation data were analyzed to obtain sample tumor mutational burden (TMB) scores. Copy number variations (CNV) frequencies of the samples were obtained from UCSC Xena (https://xena.ucsc.edu/) and the gain and loss of CNV frequencies were analyzed.

### Consensus clustering analysis based on correlated genes

Consensus clustering analysis is often used to target subgroups of one trait, which in turn allows understanding the differences between different subgroups. In our study, we performed consensus clustering analysis on TLS-RGs and co-DEGs by the “ConsensusClusterPlus” package. Kaplan-Meier (KM) curves can describe the differences in survival between different subgroups and can be performed with the “survival” package.

### Enrichment analysis

Kyoto Encyclopedia of Genes and Genomes (KEGG) enrichment analysis provides an understanding of the signaling pathways involved at the molecular level. Gene ontology (GO) enrichment analysis enables the qualification and description of gene and protein functions, including biological process (BP), cellular component (CC) and molecular function (MF). Gene set variation analysis (GSVA) enables differential analysis at the level of signaling pathways. In our study, they can be implemented by the “clusterProfiler” package and the “GSVA” package.

### Immune landscape assessment

In order to understand the immune landscape of each patient, we apply multiple algorithms to assess the immune cell infiltration and immune score. Single-sample Gene Set Enrichment Analysis (ssGSEA) is able to analyze the infiltration of 23 types of immune cells in each sample, which can be implemented through the “ssGSEA” package. The ESTIMATE package provides ESTIMATE scores, immune scores and tumor purity for each sample.

### Principal component analysis (PCA)

PCA analysis enables the assessment of the classification of different subgroups, and to a certain extent can reflect the differences between subgroups. In addition, TLS scores can be obtained by PCA analysis of TLS-RGs.

### Construction and verification of nomograms

Nomograms are widely used for prognostic prediction of tumors. In our study, nomograms based on clinical characteristics and TLS scores were constructed to predict 1-, 3-, and 5-OS of GC patients. Concordance index (C-index) and decision curves were able to assess the predictive accuracy of nomograms. Univariate and multifactorial regression analysis were used for the identification of prognostic marker genes.

### Immunotherapy response and drug sensitivity analysis

The tumor immune dysfunction and exclusion (TIDE) score allows assessment of the likelihood of tumor immune escape based on gene expression data. We obtained TIDE scores for each sample through the TIDE database (http://tide.dfci.harvard.edu/login/). The Cancer Imaging Archive (TCIA) results can reflect the outcome of patients treated with anti-PD-1 or anti-CTAL4. Microsatellite instability (MSI) is considered one of the important indicators of tumor immune response, so we further analyzed the TCIA results to obtain MSI results for each sample. Drug sensitivity analysis is performed through the “pRRophetic” package, where the half maximal inhibitory concentration (IC50) is used as an indicator of drug sensitivity.

### Statistical analysis

The data processing, analysis, and visualization involved in this study were performed by R software (version 4.1.2). Wilcoxon rank sum test and analysis of variance (ANOVA) were used for the analysis of differences between two and three groups, respectively. *p* < 0.05 was considered a statistical difference between the comparison groups.

### Data availability statement

The datasets in this study were obtained from the TCGA database (https://portal.gdc.cancer.gov/) or the GEO database (https://www.ncbi.nlm.nih.gov/geo/). Raw data and original codes are included in the article or in the supplementary material and further inquiries can be addressed to the respective authors.

## RESULTS

### Expression and mutation analysis of TLS-RGs in GC

**Figure 1:**
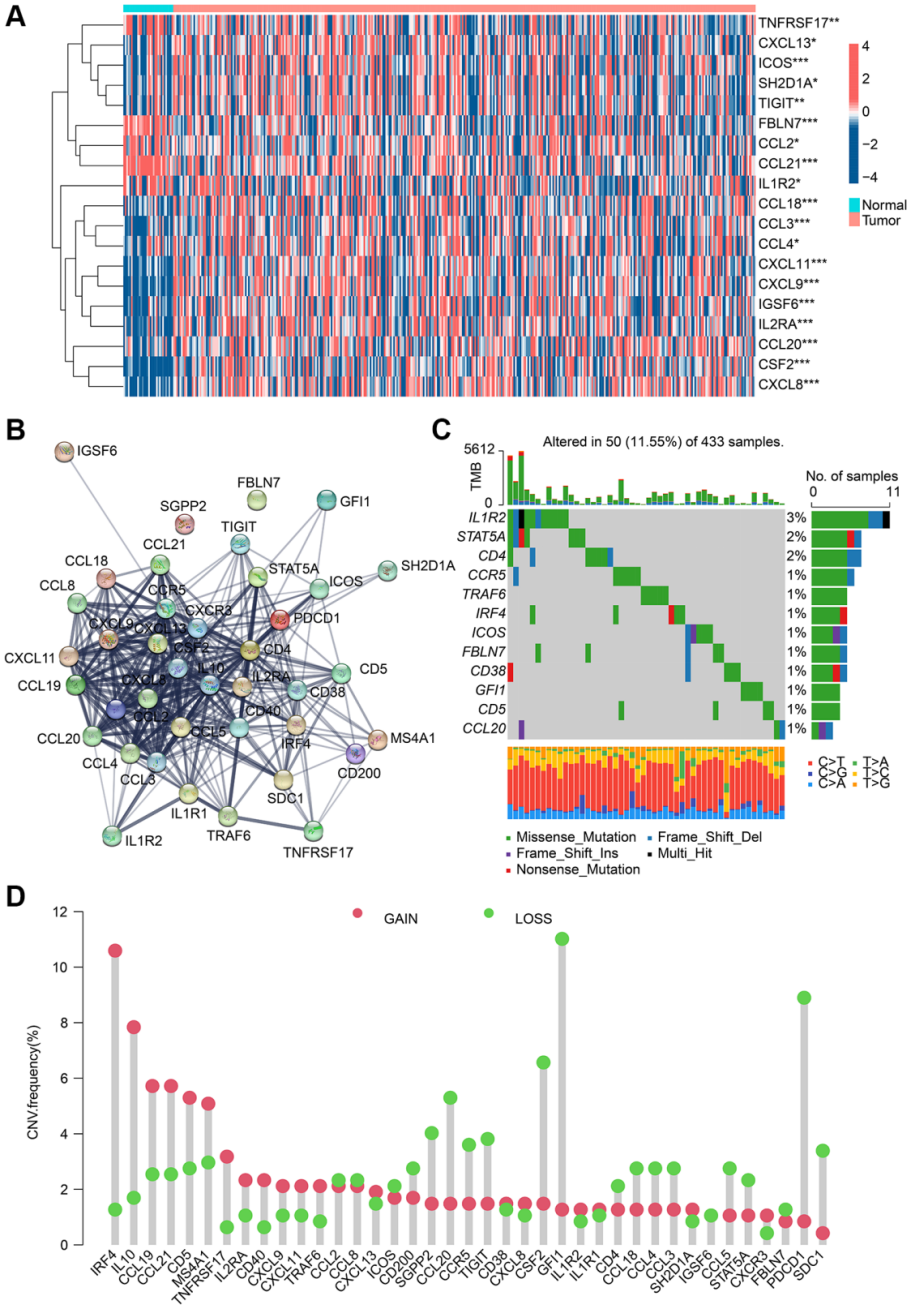
**Analysis result of TLS-RGs in expression and mutational landscape.** (**A**) Result of DE-TLS-RGs expression in normal and GC tissues. (**B**) PPI network analysis of TLS-RGs. (**C**) Somatic mutational landscape of TLS-RGs. (**D**) CNV frequencies of TLS-RGs in GC. ^*^p < 0.05; ^**^p < 0.01; ^***^p < 0.001.

According to the TCGA dataset, 39 TLS-RGs were obtained and the expression differences of these 39 TLS-RGs in normal tissue and GC tumor tissue were analyzed (Supplementary Table 1). The results are shown in Figure 1A, a total of 19 TLS-RGs were considered to have significantly altered expression in GC, 14 of which were up-regulated and 5 were down-regulated. The PPI network results indicated a significant association and interplay of TLS-RGs in GC (Figure 1B). TLS-RGs were also mutated in GC (11.55%), notably, IL1R2 (3%), STAT5A (2%), and CD4 (2%) had the highest mutation frequency in GC (Figure 1C). CNV frequency results showed that there were significant copy number changes in TLS-RGs in GC and more genes lost their copy number (Figure 1D). These results confirmed that the expression and mutation frequency of TLS-RGs were altered in GC indicating that TLS-RGs were associated with GC.

### Identification and immune infiltration of TLS subgroups in GC

**Figure 2:**
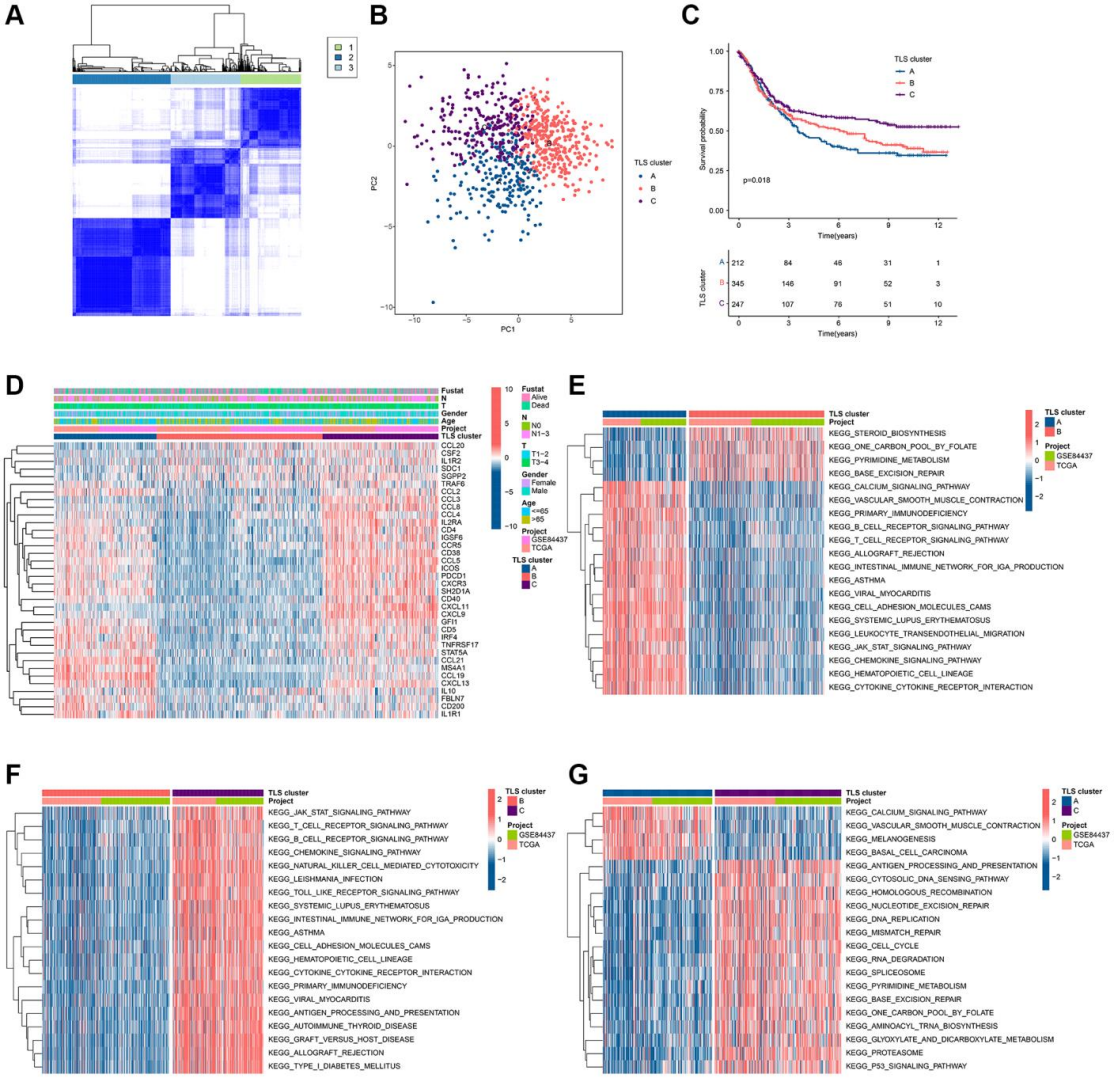
**Identification of TLS subgroups in GC.** (**A**) Results of unsupervised clustering analysis. (**B**) PCA results of three clusters. (**C**) KM curves of three clusters. (**D**) Heat map of TLS-RGs expression in three clusters. (**E**) Enrichment analysis of differential genes between cluster A and cluster B. (**F**) Enrichment analysis of differential genes between cluster A and cluster B. (**G**) Enrichment analysis of differential genes between cluster A and cluster B.

To clarify the role of TLS-RGs in GC, we performed unsupervised clustering analysis of GC patients based on the expression of 39 TLS-RGs. Good results were obtained when the number of subgroups was 3 (Figure 2A). PCA results showed the separation of the three clusters from each other (Figure 2B). The KM curves showed that the survival curves were different among three clusters, with cluster C being the best and cluster A or cluster B being worse (Figure 2C). The expression heat map of TLS-RGs in three clusters is shown in Figure 2D, and it should be noted that the expression of TLS-RGs in cluster B is the most specific. To understand the reasons for the survival differences between these three clusters, differential analysis was performed for two of these three clusters and the biological signals involved in the differentially expressed genes were identified by enrichment analysis (Figure 2D–2G). The results showed that immune-related pathways were more enriched in cluster A and cluster C, and cluster C was higher than cluster A (Figure 2D–2G). This suggests that immunity is involved in the regulatory of TLS-RGs in GC.

**Figure 3:**
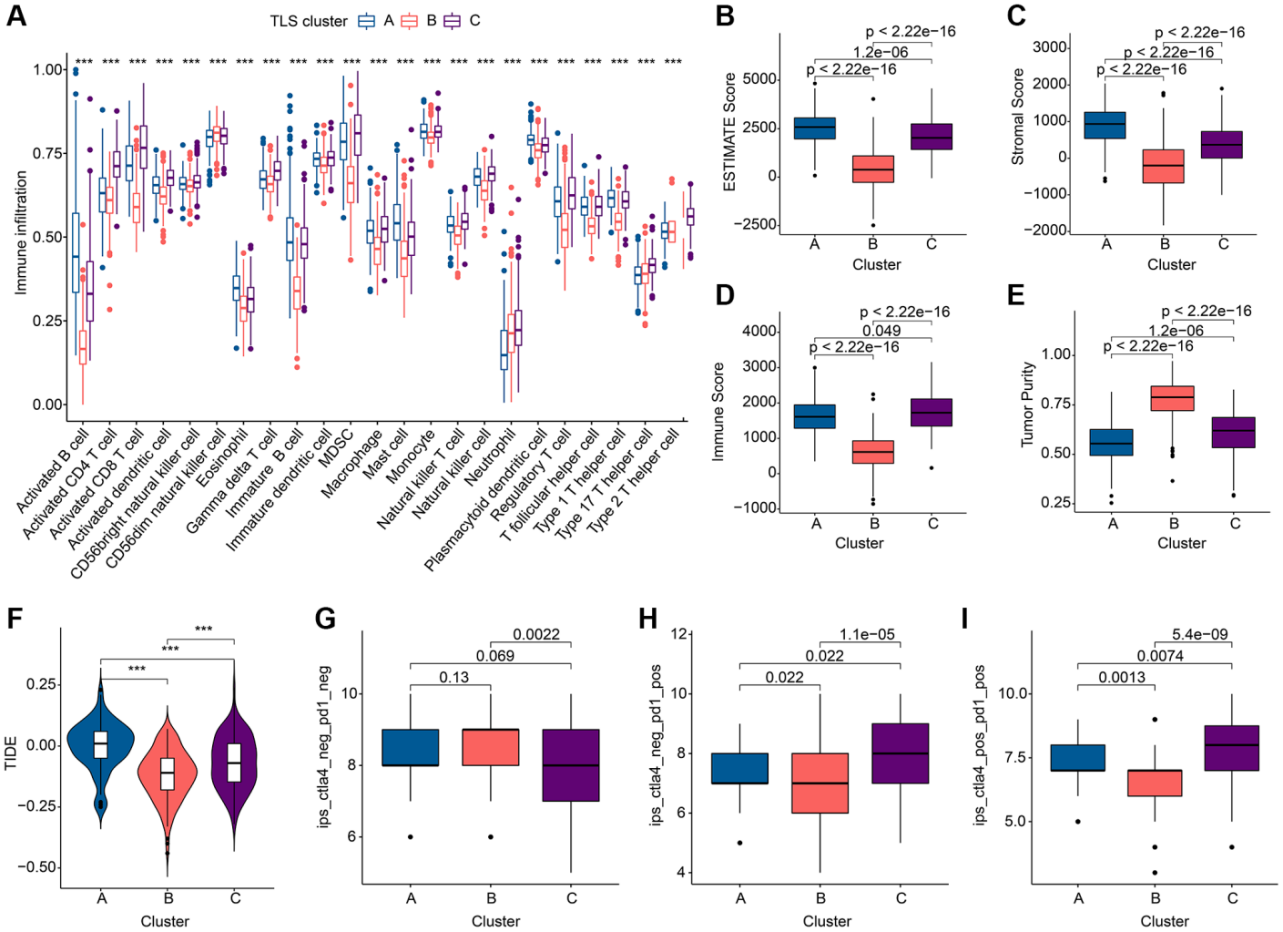
**Immune landscape analysis results of TLS subgroups in GC.** (**A**) Results of 23 immune cell abundance analyses by ssGSEA. (**B**–**E**) ESTIMATE algorithm results. (**F**) TIDE score results. (**G**–**I**). Results of immunotherapy analysis. ^*^p < 0.05; ^**^p < 0.01; ^***^p < 0.001.

To investigate the relationship between the altered immune environment and TLS subgroups, we applied multiple immune algorithms to assess the immune landscape of the samples. ssGSEA results showed significant differences in the abundance of 23 immune cell in the 3 clusters (Figure 3A). It is worth noting that the infiltration of Neutrophils and Type 17 T helper cells in TLS cluster A or TLS cluster C is different from other immune cells. And cluster C had a higher ESTIMATE score and higher immune score but lower tumor purity compared to cluster B (Figure 3B–3E). TIDE results also pointed to a better immune response in cluster C (Figure 3F). In addition, we performed immunotherapy analysis on these 3 clusters and found that cluster C showed a greater response to anti-PD-1/anti-PD-1+ anti-CTLA4 treatment (Figure 3G–3I). In conclusion, the better survival of cluster C is closely related to its own immune environment.

### Subgroup identification based on co-differential expressed genes

**Figure 4:**
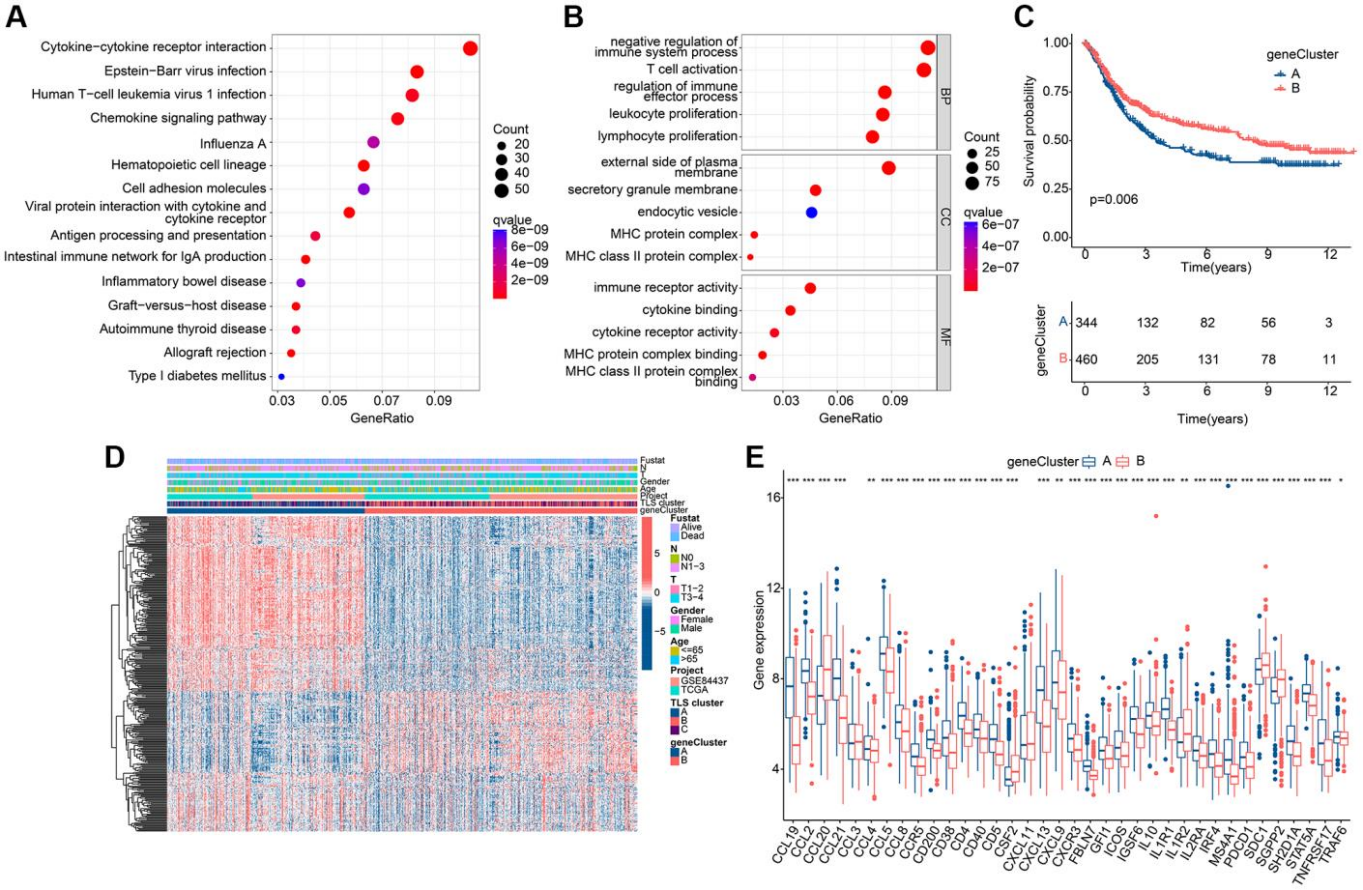
**Results of subgroup identification based on co-differentially expressed genes.** (**A**) KEGG results of co-DEGs. (**B**) GO results of co-DEGs. (**C**) KM curves of gene clusters. (**D**) Expression heat map in gene clusters of co-DEGs. (**E**) Expression differences of TLS-RGs in gene clusters. ^*^p < 0.05; ^**^p < 0.01; ^***^p < 0.001.

To garner subgroups marked by more conspicuous distinctions in prognostic outcomes, a reiteration of consensus clustering analysis was executed upon a cohort of GC patients. This secondary consensus clustering analysis was undertaken with the intention of amplifying the repertoire of DEGs, with a specific focus on identifying those that bear relevance to prognosis. The overarching objective herein was to refine the construction of a more robust prognostic risk model. Subsequently, a compendium of common DEGs was discerned within the context of TLS clusters through the adept utilization of the “limma” computational package. Thereafter, a meticulous enrichment analysis was conducted, yielding pertinent insights into the functional implications of these genes (Supplementary Table 2). These co-DEGs were found to be mainly enriched in the biological functions of the immune process (Figure 4A, 4B), suggesting that TLS-RGs-mediated alterations in the immune process are critical for the regulation of GC. Further, the role of co-DEGs in GC was further discussed by dividing patients into different genetic subgroups based on the expression of co-DEGs using a consensus clustering approach. The results showed that the optimal grouping results could be obtained when the number of subgroups was 2. The KM curves indicated that there was a significant difference in prognosis between the two gene clusters, with gene cluster B having a better prognosis than gene cluster A (Figure 4C), and the difference was greater than that of the TLS cluster (P_gene cluster_ = 0.006 < P_TLS cluster_ = 0.018). Expression heat map of co-DEGs is shown in Figure 4D, and it is clear that their expression is altered in these two gene clusters. We also analyzed the expression of TLS-RGs between these two gene clusters and the results showed that the expression of most TLS-RGs differed and that the expression of TLS-RGs was higher in gene cluster A than in gene cluster B (Figure 4E). This indicates that TLS-RGs in gene clusters appear significantly different.

### Assessment and classification of TLS scores

**Figure 5:**
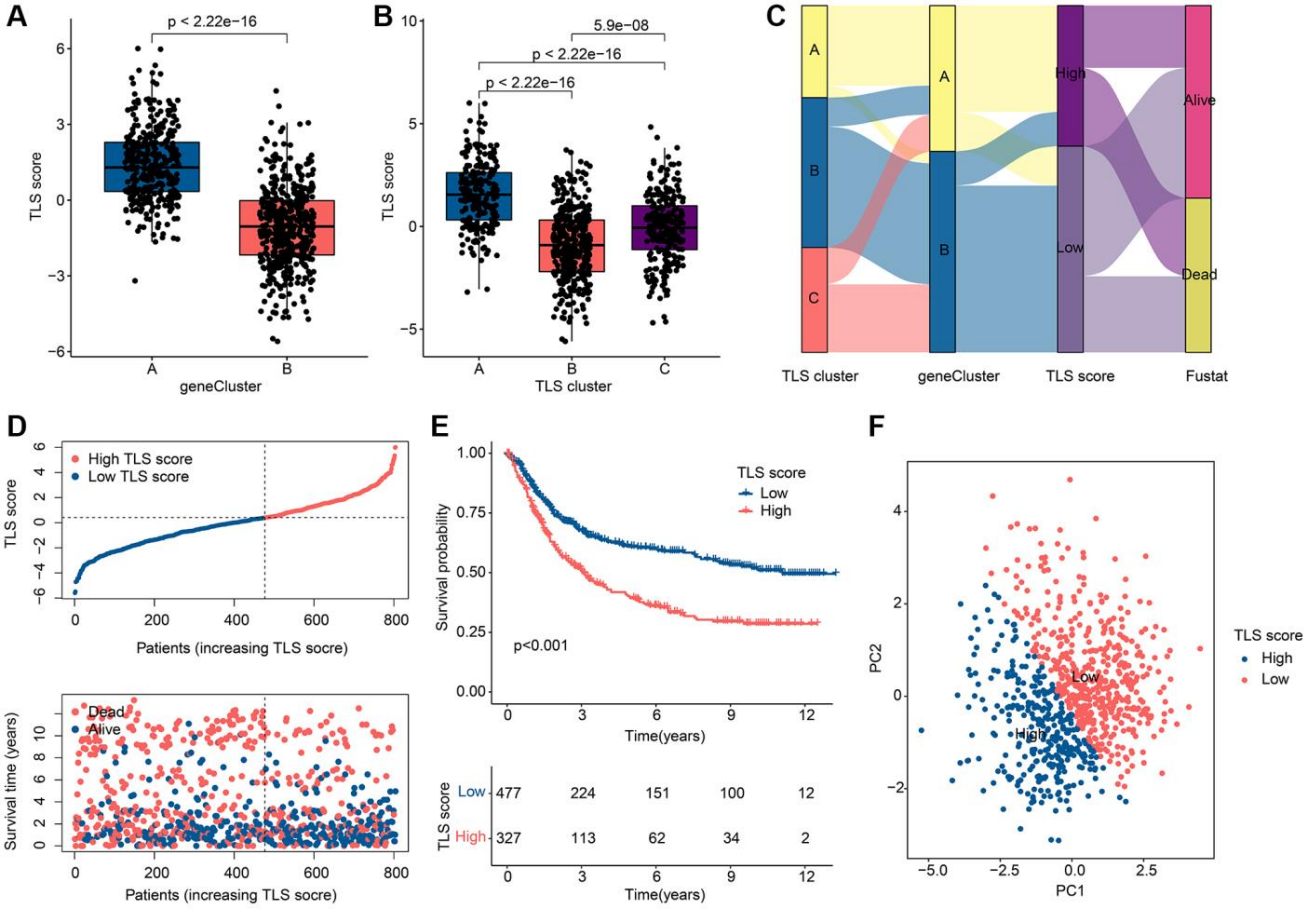
**Assessment and classification results of TLS scores.** (**A**) Differences in TLS scores among gene clusters. (**B**) Differences in TLS scores among TLS clusters. (**C**) Distribution of TLS scores among gene clusters, TLS clusters and TLS score subgroups. (**D**) Scatter plot of GC patients with different TLS scores. (**E**) KM curve results of GC patients with different TLS scores. (**F**) PCA results of GC patients with different TLS scores. ^*^p < 0.05; ^**^p < 0.01; ^***^p < 0.001.

In pursuit of enhanced precision in forecasting both OS and treatment responsiveness among GC patients, a TLS scoring framework was meticulously crafted, leveraging the outcomes of Principal Component Analysis (PCA) applied to gene clusters. This strategic development seeks to furnish a robust predictive tool capable of furnishing more refined prognostic and treatment response insights for the benefit of GC patient care. As shown in Figure 5A, 5B, TLS scores differed in either gene clusters or TLS clusters. Note that TLS scores are highest in gene cluster A and TLS cluster A, which indicates that TLS scores are associated with immune regulation. Based on the TLS score, the optimal threshold was selected and the patients were divided into a low TLS score group and a high TLS score group. The distribution of GC patients among TLS clusters, gene clusters and TLS score clusters is shown in Figure 5C. The scatter plot showed that GC patients in the low TLS score group had a longer survival time (Figure 5D). GC patients in the low TLS score group tended to have better OS (Figure 5E). PCA results showed that patients with different TLS scores were separated from each other (Figure 5F).

### Construction of nomogram based on clinical characteristics and TLS scores

**Figure 6:**
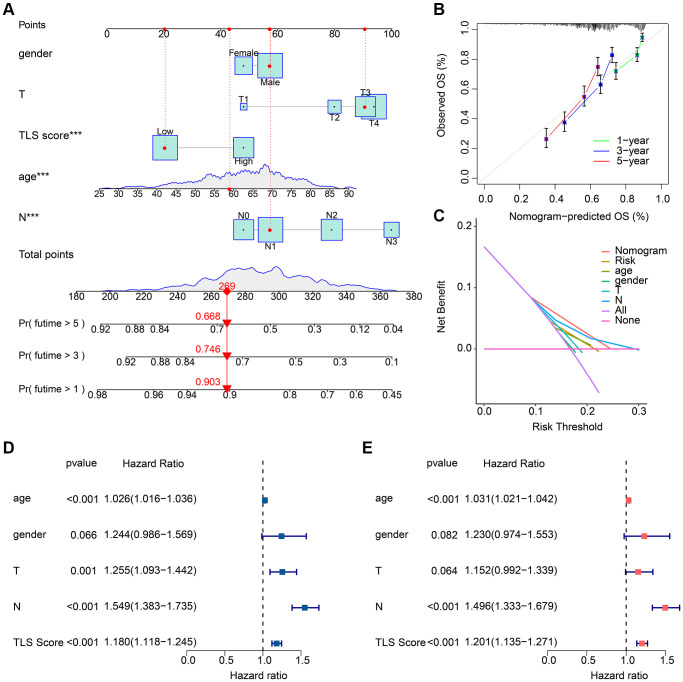
**Results of nomogram based on clinical characteristics and TLS scores.** (**A**) Prognostic nomogram based on clinical characteristics and TLS scores. (**B**) C-index results of the prognostic nomogram. Result is close to 45° indicating that the nomogram prediction is accurate. (**C**) Decision curve results of the prognostic nomogram. (**D**) Results of univariate Cox regression analysis of clinical characteristics and TLS scores. (**E**) Results of multifactorial Cox regression analysis of clinical characteristics and TLS scores. ^*^p < 0.05; ^**^p < 0.01; ^***^p < 0.001.

In order to accurately predict the prognosis of GC patients, we constructed a prognostic nomogram based on clinical characteristics and TLS scores (Figure 6A). Concordance index (C-index) results showed that the nomogram had good prognostic ability (Figure 6B). The decision curves showed that the nomogram was more accurate in predicting OS of GC patients than other clinical characteristics or TLS scores (Figure 6C). We further assessed the predictive independence of clinical characteristics and TLS scores by univariate and multifactorial Cox regression. The results showed that age (hazard ratio (HR) = 1.026, *p* < 0.001), T (HR = 1.255, *p* = 0.001), N (HR = 1.549, *p* < 0.001) and TLS score (HR = 1.180, *p* < 0.001) were associated with OS in GC patients, whereas age (HR = 1.031, *p* < 0.001), N (HR = 1.496, *p* < 0.001) and TLS score (HR = 1.201, *p* < 0.001) were able to serve as independent prognostic factors for GC patients (Figure 6D, 6E).

### Immune landscape and immunotherapy in different TLS scoring groups

**Figure 7:**
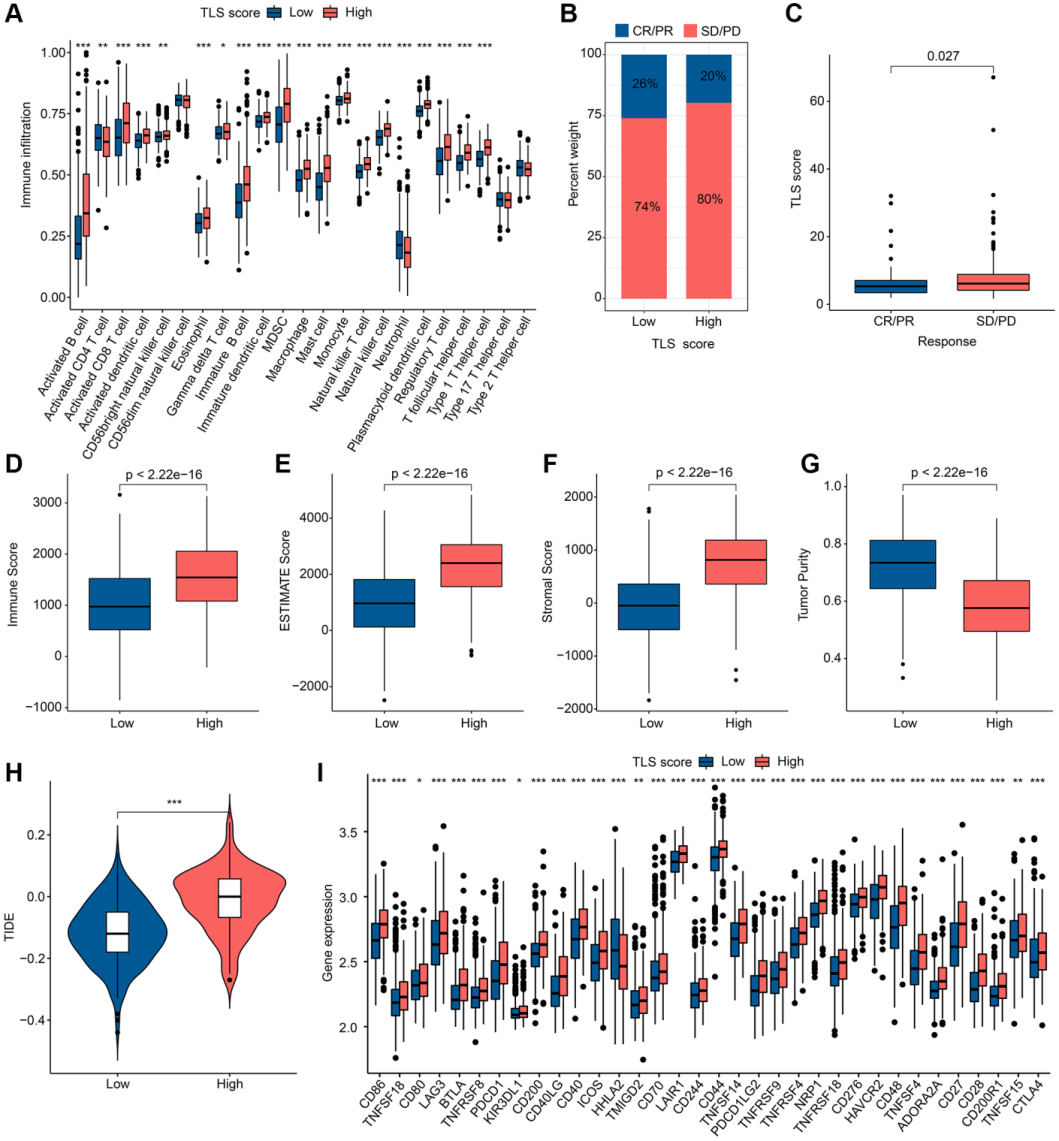
**Results of immune landscape and immunotherapy in different TLS score groups.** (**A**) Abundance results of 23 immune cells by ssGSEA in different TLS score groups. (**B**) Results of anti-PD-L1 immunotherapy responses in different TLS score groups; Abbreviations: CR: complete remission; PR: partial remission; SD: stable disease; PD: progressive disease. (**C**) Distribution of TLS scores in different immunotherapy responses. (**D**–**G**) ESTIMATE results in different TLS score groups. (**H**) TIDE results in different TLS score groups. (**I**) Results of ICP in different TLS score groups. ^*^p < 0.05; ^**^p < 0.01; ^***^p < 0.001.

The abundance of 23 immune cells in the TLS score subgroups was analyzed by ssGSEA, and the results showed that there were significant differences in the distribution of the other 20 immune cells in different TLS scoring groups, except for CD56dim natural killer cell, Type 17 T helper cell and Type 2 T helper cell (Figure 7A). As shown in Figure 7B, 7C, SD/PD group had higher TLS scores. ESTIMATE results indicated that the high TLS score group had higher ESTIMATE score, immune score as well as lower tumor purity (Figure 7D–7G). Notably, there was more potential for immune escape in the high TLS score group due to the higher TIDE score in the high TLS score group compared to the low TLS score group (Figure 7H). In the analysis of ICP, we found that the expression of ICP was higher in the high TLS score group than in the low TLS score group, with the exception of HHLA2 (Figure 7I). In conclusion, we found significant immunological differences between subgroups of TLS score groups, with patients in the low TLS score group receiving immunotherapy with better outcomes than the high TLS score group.

### Analysis of tumor mutation landscape and drug sensitivity in different TLS score groups

**Figure 8:**
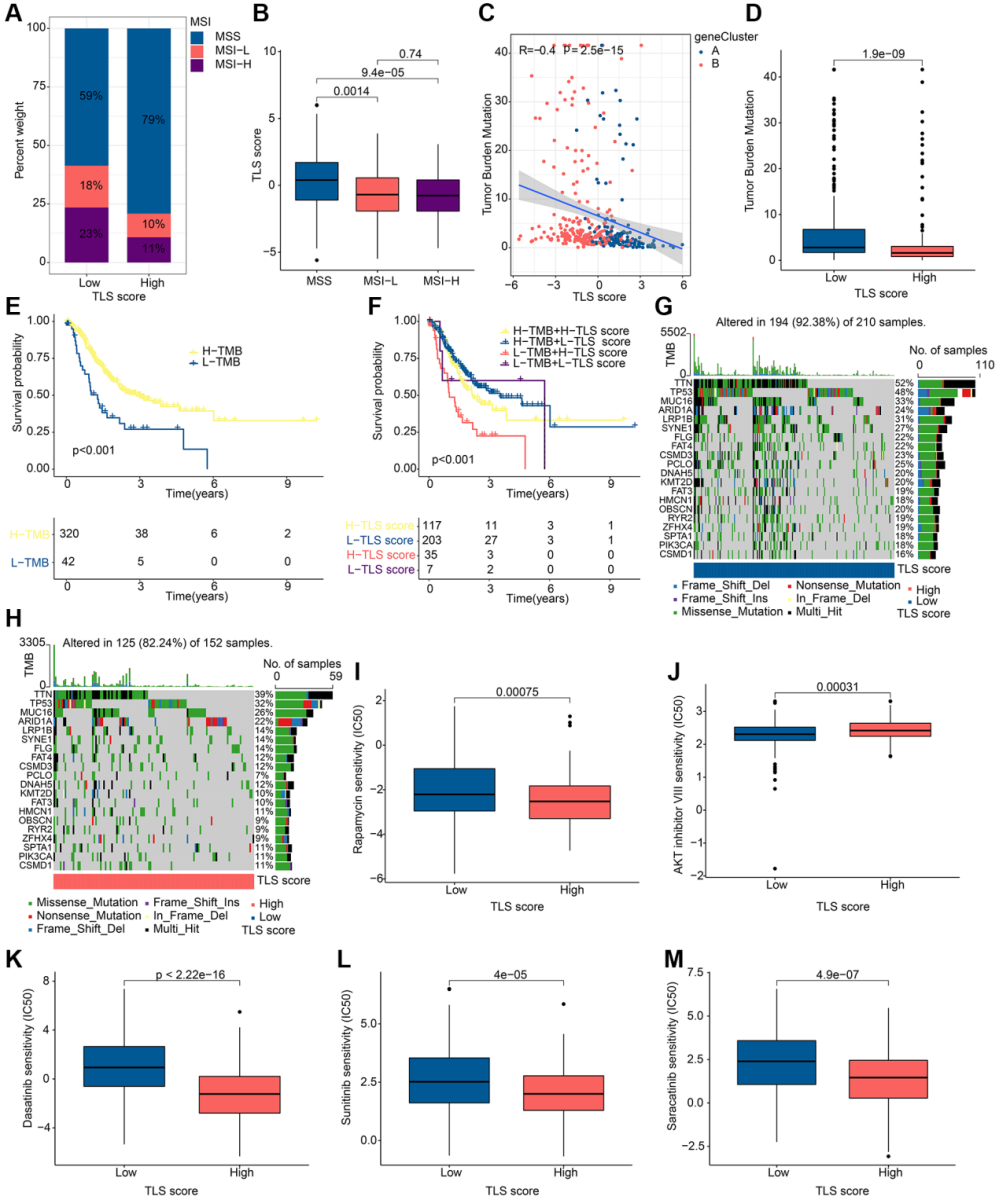
**Results of tumor mutation landscape and drug sensitivity analysis in different TLS score groups.** (**A**) MSI results in different TLS score groups. (**B**) Correlation results of MSI and TLS score. (**C**) Correlation results of TMB and TLS score. (**D**) Distribution of TMB in different TLS score groups. (**E**) KM curves of patients with different TMB. (**F**) KM curves of patients with different TMB and different TLS scores. (**G**) Somatic mutation analysis results in low TLS scores. (**H**) Somatic mutation analysis results in high TLS scores. (**I**–**M**) Drug sensitivity analysis results in different TLS score groups. ^*^p < 0.05; ^**^p < 0.01; ^***^p < 0.001.

Microsatellite Instability (MSI) is considered as one of the important indicators of tumor immune response. In our study, patients in the low TLS score group were associated with MSI-L and MSI-H, while patients in the high TLS score group were associated with MISS (Figure 8A, 8B). Tumor mutation burden (TMB) can help predict patient response to immunotherapy. In our study, TMB was negatively correlated with TLS score (Figure 8C). Notably, TMB was higher in the low TLS score group than in the high TLS score group (Figure 8D), indicating that patients in the low TLS score group had a better immunotherapy response. Subsequently, we performed survival analysis on patients with different TMB, and the results showed that patients with high TMB tended to have better OS (Figure 8E). Further combined with the TLS score, patients with low TLS score accompanied by high TMB had the best prognosis (Figure 8F). The results of somatic mutation analysis for TLS score showed that the incidence of mutations in TTM, TP53, MUC16 and ARID1A were all higher than 20%, and it should be noted that the incidence of mutations in these genes was higher in low TLS score than in high TLS score (Figure 8G, 8H). These results suggest that TLS-RGs can influence the response to tumor immunotherapy. Drug sensitivity can reflect the patient’s response to drug therapy, and drug sensitivity analysis for TLS score was performed in our experiments. The results showed that Rapamycin, Dasatinib, Sunitinib and Saracatinib had a higher IC50 in the low TLS score group of patients, in contrast to AKT inhibitor VII (Figure 8I–8M). In conclusion, TLS-RGs was also associated with drug sensitivity in GC patients.

## DISCUSSION

As one of the most common tumors worldwide, gastric cancer occupies the fourth place in the tumor incidence and the fifth place in the mortality rate of all tumors [24]. The treatment of tumors is largely related to the staging of tumors, however the highly heterogeneous nature of GC has led to more challenges facing traditional staging [25]. Therefore, there is an urgent need to focus on more factors to guide the staging of GC and provide personalized treatment for GC patients. TLS as a factor associated with tumorigenesis and prognosis, is gradually demonstrating its importance [26]. In particular, TLS may play a direct role in the tumor immune response [27]. Therefore, a comprehensive analysis of the role of TLS in GC may be able to overcome the poor outcome due to the high heterogeneity of GC patients.

In this study, we found that most TLS-RGs were altered in GC patients in terms of expression and variation, especially IL-1R2, STAT5A and CD4 with higher mutated frequency. Interleukin-1 receptor type II (IL-1R2), as a negative regulator of the IL-1 system, plays a key role in many inflammatory diseases and cancers [28]. The signal transducer and activator of transcription 5A (STAT5A) can influence the major regulators of cell growth and regulate tumorigenesis [29]. CD4 is mainly expressed by helper T cells and has a crucial role in human immunity [30]. These genes, once altered, may be able to influence tumorigenesis, immune landscape, and immune escape.

Based on TLS-RGs, we divided GC into three TLS clusters. It is worth noting that differences in immune pathways may be a key factor influencing the prognosis of different TLS clusters, since in our study immune pathways in TLS cluster C were found to be enriched and accompanied by an optimal prognosis. Differences in the infiltration of immune cells can often influence the prognosis of GC patients [31]. In our study, both TLS cluster A and TLS cluster C had high immune scores, but their prognosis was very different, especially TLS cluster A had a higher degree of immune escape. By ssGSEA analysis, we found that the proportion of Neutrophils and Type 17 T helper cells differed significantly in TLS cluster A and TLS cluster C. Perhaps they are the key cells affecting GC immune escape.

Based on the co-DEGs between the TLS clusters, we divided GC patients into two gene clusters and obtained TLS scores by PCA analysis. Thereafter optimal threshold was screened and GC patients were divided into different TLS score groups. Notably, the high TLS score group had a worse prognosis. Prognostic nomogram with good predictive accuracy was constructed. Cox regression analysis showed that TLS score was an independent prognostic factor for GC patients. Similarly, we found differences in immunity between the different TLS score groups. Cancer growth and progression are directly associated with suppression of the immune system, in which suppressive immune checkpoints play a crucial role [32]. We found that most ICP differed among different TLS score groups, especially with increased expression in the high TLS score group. The low TLS score group induced antitumor immune responses and inhibited tumor immune escape resulting in better response to immunotherapy. We also analyzed the sensitivity of different TLS score groups to potential drugs. The results revealed that Rapamycin, Dasatinib, Sunitinib and Saracatinib had high IC50 in the low TLS score group, while AKT inhibitor VII had high IC50 in the high TLS score group. It indicates that patients with different TLS scores have different sensitivity to chemotherapy, and we can rely on TLS scores to personalize the treatment for GC patients.

This study has been predicated upon an analysis of publicly available datasets, bearing the inherent limitation of lacking sample validation. Nevertheless, we plan to validate our signature in gastric cancer in future studies. Further experimental validation is needed regarding the exact regulatory mechanism of TLS RGS in GC.

## CONCLUSION

In summary, we performed a comprehensive analysis of the role of TLS-RGs in GC, indicating that TLS-RGs are able to serve as biomarkers for the predictive of GC patients’ prognosis. TLS-RGs also influences and embodies the immune landscape of GC patients, which in turn can predict the outcome of patients receiving immunotherapy or chemotherapy and provide a new reference basis for personalized treatment of GC patients.

## Supplementary Materials

Supplementary Table 1

Supplementary Table 2

## References

[1] Karimi P, Islami F, Anandasabapathy S, Freedman ND, Kamangar F. Gastric cancer: descriptive epidemiology, risk factors, screening, and prevention. Cancer Epidemiol Biomarkers Prev. 2014; 23:700–13. 10.1158/1055-9965.EPI-13-1057 24618998 PMC4019373

[2] Sheikh IA, Mirza Z, Ali A, Aliev G, Ashraf GM. A proteomics based approach for the identification of gastric cancer related markers. Curr Pharm Des. 2016; 22:804–11. 10.2174/1381612822666151209151848 26648471

[3] Lordick F, Carneiro F, Cascinu S, Fleitas T, Haustermans K, Piessen G, Vogel A, Smyth EC, and ESMO Guidelines Committee. Gastric cancer: ESMO Clinical Practice Guideline for diagnosis, treatment and follow-up. Ann Oncol. 2022; 33:1005–20. 10.1016/j.annonc.2022.07.004 35914639

[4] Wang Z, Han W, Xue F, Zhao Y, Wu P, Chen Y, Yang C, Gu W, Jiang J. Nationwide gastric cancer prevention in China, 2021-2035: a decision analysis on effect, affordability and cost-effectiveness optimisation. Gut. 2022; 71:2391–400. 10.1136/gutjnl-2021-325948 35902213

[5] Chen QY, Zhong Q, Liu ZY, Huang XB, Que SJ, Zheng WZ, Li P, Zheng CH, Huang CM. Advances in laparoscopic surgery for the treatment of advanced gastric cancer in China. Eur J Surg Oncol. 2020; 46:e7–13. 10.1016/j.ejso.2020.07.015 32709375

[6] Correa P. Gastric cancer: overview. Gastroenterol Clin North Am. 2013; 42:211–7. 10.1016/j.gtc.2013.01.002 23639637 PMC3995345

[7] Machlowska J, Baj J, Sitarz M, Maciejewski R, Sitarz R. Gastric Cancer: Epidemiology, Risk Factors, Classification, Genomic Characteristics and Treatment Strategies. Int J Mol Sci. 2020; 21:4012. 10.3390/ijms21114012 32512697 PMC7312039

[8] Gao JP, Xu W, Liu WT, Yan M, Zhu ZG. Tumor heterogeneity of gastric cancer: From the perspective of tumor-initiating cell. World J Gastroenterol. 2018; 24:2567–81. 10.3748/wjg.v24.i24.2567 29962814 PMC6021770

[9] Im SA, Kim JW, Kim JS, Kim MA, Jordan B, Pickl M, Han SW, Oh DY, Lee HJ, Kim TY, Kim WH, Yang HK, Bang YJ. Clinicopathologic characteristics of patients with stage III/IV (M(0)) advanced gastric cancer, according to HER2 status assessed by immunohistochemistry and fluorescence in situ hybridization. Diagn Mol Pathol. 2011; 20:94–100. 10.1097/PDM.0b013e3181fc02b7 21532492

[10] Patel TH, Cecchini M. Targeted Therapies in Advanced Gastric Cancer. Curr Treat Options Oncol. 2020; 21:70. 10.1007/s11864-020-00774-4 32725377

[11] Pimenta EM, Barnes BJ. Role of Tertiary Lymphoid Structures (TLS) in Anti-Tumor Immunity: Potential Tumor-Induced Cytokines/Chemokines that Regulate TLS Formation in Epithelial-Derived Cancers. Cancers (Basel). 2014; 6:969–97. 10.3390/cancers6020969 24762633 PMC4074812

[12] Dieu-Nosjean MC, Goc J, Giraldo NA, Sautès-Fridman C, Fridman WH. Tertiary lymphoid structures in cancer and beyond. Trends Immunol. 2014; 35:571–80. 10.1016/j.it.2014.09.006 25443495

[13] Lin L, Hu X, Zhang H, Hu H. Tertiary Lymphoid Organs in Cancer Immunology: Mechanisms and the New Strategy for Immunotherapy. Front Immunol. 2019; 10:1398. 10.3389/fimmu.2019.01398 31281318 PMC6596321

[14] Dieu-Nosjean MC. Tumor-Associated Tertiary Lymphoid Structures: A Cancer Biomarker and a Target for Next-generation Immunotherapy. Adv Exp Med Biol. 2021; 1329:51–68. 10.1007/978-3-030-73119-9_3 34664233

[15] Ding GY, Ma JQ, Yun JP, Chen X, Ling Y, Zhang S, Shi JY, Chang YQ, Ji Y, Wang XY, Tan WM, Yuan KF, Yan B, et al. Distribution and density of tertiary lymphoid structures predict clinical outcome in intrahepatic cholangiocarcinoma. J Hepatol. 2022; 76:608–18. 10.1016/j.jhep.2021.10.030 34793865

[16] Horeweg N, Workel HH, Loiero D, Church DN, Vermij L, Léon-Castillo A, Krog RT, de Boer SM, Nout RA, Powell ME, Mileshkin LR, MacKay H, Leary A, et al, and TransPORTEC consortium. Tertiary lymphoid structures critical for prognosis in endometrial cancer patients. Nat Commun. 2022; 13:1373. 10.1038/s41467-022-29040-x 35296668 PMC8927106

[17] Peng Y, Xiao L, Rong H, Ou Z, Cai T, Liu N, Li B, Zhang L, Wu F, Lan T, Lin X, Li Q, Ren S, et al. Single-cell profiling of tumor-infiltrating TCF1/TCF7^+^ T cells reveals a T lymphocyte subset associated with tertiary lymphoid structures/organs and a superior prognosis in oral cancer. Oral Oncol. 2021; 119:105348. 10.1016/j.oraloncology.2021.105348 34044317

[18] Barmpoutis P, Di Capite M, Kayhanian H, Waddingham W, Alexander DC, Jansen M, Kwong FNK. Tertiary lymphoid structures (TLS) identification and density assessment on H&E-stained digital slides of lung cancer. PLoS One. 2021; 16:e0256907. 10.1371/journal.pone.0256907 34555057 PMC8460026

[19] Dai S, Zeng H, Liu Z, Jin K, Jiang W, Wang Z, Lin Z, Xiong Y, Wang J, Chang Y, Bai Q, Xia Y, Liu L, et al. Intratumoral CXCL13^+^CD8^+^T cell infiltration determines poor clinical outcomes and immunoevasive contexture in patients with clear cell renal cell carcinoma. J Immunother Cancer. 2021; 9:e001823. 10.1136/jitc-2020-001823 33589528 PMC7887366

[20] Jahanafrooz Z, Mosafer J, Akbari M, Hashemzaei M, Mokhtarzadeh A, Baradaran B. Colon cancer therapy by focusing on colon cancer stem cells and their tumor microenvironment. J Cell Physiol. 2020; 235:4153–66. 10.1002/jcp.29337 31647128

[21] Wang F, Wei XL, Wang FH, Xu N, Shen L, Dai GH, Yuan XL, Chen Y, Yang SJ, Shi JH, Hu XC, Lin XY, Zhang QY, et al. Safety, efficacy and tumor mutational burden as a biomarker of overall survival benefit in chemo-refractory gastric cancer treated with toripalimab, a PD-1 antibody in phase Ib/II clinical trial NCT02915432. Ann Oncol. 2019; 30:1479–86. 10.1093/annonc/mdz197 31236579 PMC6771223

[22] Schumacher TN, Thommen DS. Tertiary lymphoid structures in cancer. Science. 2022; 375:eabf9419. 10.1126/science.abf9419 34990248

[23] Cabrita R, Lauss M, Sanna A, Donia M, Skaarup Larsen M, Mitra S, Johansson I, Phung B, Harbst K, Vallon-Christersson J, van Schoiack A, Lövgren K, Warren S, et al. Tertiary lymphoid structures improve immunotherapy and survival in melanoma. Nature. 2020; 577:561–5. 10.1038/s41586-019-1914-8 31942071

[24] Lei ZN, Teng QX, Tian Q, Chen W, Xie Y, Wu K, Zeng Q, Zeng L, Pan Y, Chen ZS, He Y. Signaling pathways and therapeutic interventions in gastric cancer. Signal Transduct Target Ther. 2022; 7:358. 10.1038/s41392-022-01190-w 36209270 PMC9547882

[25] Amin MB, Greene FL, Edge SB, Compton CC, Gershenwald JE, Brookland RK, Meyer L, Gress DM, Byrd DR, Winchester DP. The Eighth Edition AJCC Cancer Staging Manual: Continuing to build a bridge from a population-based to a more "personalized" approach to cancer staging. CA Cancer J Clin. 2017; 67:93–9. 10.3322/caac.21388 28094848

[26] Sautès-Fridman C, Lawand M, Giraldo NA, Kaplon H, Germain C, Fridman WH, Dieu-Nosjean MC. Tertiary Lymphoid Structures in Cancers: Prognostic Value, Regulation, and Manipulation for Therapeutic Intervention. Front Immunol. 2016; 7:407. 10.3389/fimmu.2016.00407 27752258 PMC5046074

[27] Munoz-Erazo L, Rhodes JL, Marion VC, Kemp RA. Tertiary lymphoid structures in cancer - considerations for patient prognosis. Cell Mol Immunol. 2020; 17:570–5. 10.1038/s41423-020-0457-0 32415259 PMC7264315

[28] Yuan M, Wang L, Huang H, Li Y, Zheng X, Shao Q, Jiang J. IL-1R2 expression in human gastric cancer and its clinical significance. Biosci Rep. 2021; 41:BSR20204425. 10.1042/BSR20204425 33704402 PMC8011276

[29] Chen X, Wang Z, Zhao X, Zhang L, Zhou L, Li X, Ge C, Zhao F, Chen T, Xie H, Cui Y, Tian H, Li H, et al. STAT5A modulates CDYL2/SLC7A6 pathway to inhibit the proliferation and invasion of hepatocellular carcinoma by targeting to mTORC1. Oncogene. 2022; 41:2492–504. 10.1038/s41388-022-02273-2 35314791

[30] Choi A, Jung YW, Choi H. The extrinsic factors important to the homeostasis of allergen-specific memory CD4 T cells. Front Immunol. 2022; 13:1080855. 10.3389/fimmu.2022.1080855 36591273 PMC9798121

[31] Ren F, Zhao Q, Zhao M, Zhu S, Liu B, Bukhari I, Zhang K, Wu W, Fu Y, Yu Y, Tang Y, Zheng P, Mi Y. Immune infiltration profiling in gastric cancer and their clinical implications. Cancer Sci. 2021; 112:3569–84. 10.1111/cas.15057 34251747 PMC8409427

[32] Alemohammad H, Najafzadeh B, Asadzadeh Z, Baghbanzadeh A, Ghorbaninezhad F, Najafzadeh A, Safarpour H, Bernardini R, Brunetti O, Sonnessa M, Fasano R, Silvestris N, Baradaran B. The importance of immune checkpoints in immune monitoring: A future paradigm shift in the treatment of cancer. Biomed Pharmacother. 2022; 146:112516. 10.1016/j.biopha.2021.112516 34906767

