# Supplementary material for: Comprehensive analysis of tertiary lymphoid structures-related genes for prognostic prediction, molecular subtypes and immune infiltration in gastric cancer
Source: Aging (Albany NY). 2023 Nov 27;15(22):13368–83. doi: 10.18632/aging.205247 (PMC10713392; doi:10.18632/aging.205247)
Supplement: Supplementary Table 1 [file aging-15-205247-s001.pdf]

SUPPLEMENTARY TABLE

Supplementary Table 1. The 39 TLS-RGs obtained from the TCGA dataset.

CCL18
CCL19
CCL2
CCL20
CCL21
CCL3
CCL4
CCL5
CCL8
CCR5
CD200
CD38
CD4
CD40
CD5
CSF2
CXCL11
CXCL13
CXCL8
CXCL9
CXCR3
FBLN7
GFI1
ICOS
IGSF6
IL10
IL1R1
IL1R2
IL2RA
IRF4
MS4A1
PDCD1
SDC1
SGPP2
SH2D1A
STAT5A
TIGIT
TNFRSF17
TRAF6
